# Correlations between Endoscopic and Histopathological Assessment of *Helicobacter pylori*-Induced Gastric Pathology—A Cross-Sectional Retrospective Study

**DOI:** 10.3390/life12122096

**Published:** 2022-12-13

**Authors:** Cătălina Dănilă, Ioana Alexandra Cardos, Andrea Pop-Crisan, Felicia Marc, Anica Hoza, Razvan Chirla, Andrei Pascalău, Calin Magheru, Simona Cavalu

**Affiliations:** Faculty of Medicine and Pharmacy, University of Oradea, 410073 Oradea, Romania

**Keywords:** *H. pylori*, atrophic gastritis, histopathology, intestinal metaplasia, RUT

## Abstract

*Helicobacter pylori* (*H. pylori*) infects about half of the world’s population and can lead to premalignant lesions and gastric cancer. Updated data about the correlation of histopathological diagnostics with endoscopic diagnostics are scarce. The objective of this study was to identify the concordance between endoscopic and histopathologic findings, with a focus on premalignant lesions. We performed a cross sectional, retrospective study over a 4-year period (2017–2021) on adult patients with dyspeptic symptoms and positive RUT (rapid urease test) in a single hospital centre, with a total of 133 patients infected with *H. pylori* being included in the study. Statistical associations between endoscopic appearance and histopathological results were found for atrophic antral gastritis (*p* = 0.001), intestinal metaplasia of the antrum (*p* = 0.018), gastric polyps (*p* < 0.001) and gastric corpus cancer (*p* = 0.012). Females were more likely to be diagnosed through endoscopy with gastric atrophy or intestinal metaplasia (*p* = 0.031), while chronic atrophic gastritis in corpus was more prevalent in patients older than 65 (*p* = 0.024). Overall, our study reveals only 21% concordance between Giemsa stain and RUT, highlighting the importance of combining rapid testing with endoscopic and histopathological diagnostic methods for a more accurate early diagnosis and prevention of gastric cancer.

## 1. Introduction

*Helicobacter pylori* (*H. pylori*) is a major risk factor for gastric cancer, being the etiological factor for 90% of MALT (mucosa-associated lymphoid tissue) lymphomas. Ever since 1994, *H. pylori* has been considered a grade I cancer risk factor by the International Agency of Cancer research [1].

It was also demonstrated that an adequate eradication therapy decreased the incidence of MALT lymphomas and induced regression among patients diagnosed with this pathology [2]. It has a fecal–oral transmission method, with high-incidence in developing countries where water sources tend to be more polluted and poor hygiene is present [3].

*H. pylori* is an infection with a high prevalence rate. A review study published by Zamani et al. in 2018 [4] conducted on 73 countries and 6 continents found that the overall prevalence of the infection is 44.3% (95% CI: 40.9–47.7), varying considerably between geographic area. Thus, in developing countries the infection rate is 50.8% (95% CI: 46.8–54.7), while in developed countries the infection rate is 34.7% (95% CI: 30.2–39.3). The study also showed that the prevalence is higher in men (95% CI: 42.1–50.5) than in women (95% CI: 39–46.5). In Romania, the prevalence of the infection is yet to be studied, while the complex data is missing. To date, only one study has been conducted in 2017, in Craiova (southern Romania) on 1525 dyspeptic patients, with the prevalence of *H. pylori* infection being 63.67% [5].

The most important premalignant lesions in gastric carcinogenesis are gastric atrophy and intestinal metaplasia. The bacterium *H. pylori*, the host and the environmental variables all influence the severity of stomach mucosal inflammation.

A rarefaction of gastric glands characterizes gastric atrophy. It can range from mild atrophy (a reduction of less than a third of the gland volume) to severe atrophy (a reduction of two-thirds of the gland volume). The volume of the glands is determined by the volume of the lamina propria, which can be expanded by lymphoplasmacytic element infiltration, especially when there is an associated inflammation. As a result, in chronic active gastritis, determining gland volume is challenging and atrophy is overestimated [6]. The replacement of gastric mucosa by intestinal mucosa is known as intestinal metaplasia. This lesion occurs later in time than stomach atrophy [7,8].

Gastric ulcers develop in 10–20% of infected individuals, and 1–3% may develop gastric cancer [9]. The risk of gastric cancer is 3–6-fold higher in *H. pylori*-infected individuals, compared to non-infected individuals [10,11].

In the present context, the aim of this observational study was to identify the demographic characteristics of the *H. pylori*-infected patients (age, gender, place of provenance) and to determine the gastric pathologies induced by the infection. The second objective was to correlate the endoscopic and histopathological findings and to evaluate the concordance between RUT and Giemsa stain for the diagnosis of *H. pylori*.

## 2. Materials and Methods

### 2.1. Patient Characteristics

This retrospective study was conducted over a 4-year period (2017–2021) in the County Emergency Clinical Hospital Oradea, Romania, on 133 patients admitted in the Digestive Endoscopy Department, that underwent upper gastrointestinal endoscopy with stomach biopsy and had a positive RUT for *H. pylori* infection. The study was conducted according to the ethical guidelines of the Declaration of Helsinki and approved by the institutional board of the County Emergency Clinical Hospital Oradea, Romania, no. 47778/16.12.2021.

The criteria for inclusion were: patients over 18 years old with symptomatic dyspeptic syndrome (such as epigastric pain, pyrosis, nausea), that had a positive urease test for *H. pylori* infection and underwent upper gastrointestinal endoscopy with biopsies.

All patients were examined by a clinician and subjected to routinely available blood tests: blood cell count, liver and renal function tests, coagulation profile. Patients that were administered proton-pump inhibitors, H2 antagonists, bismuth agents or antibiotics 4 weeks prior were not included in the study.

### 2.2. Endoscopy

All patients underwent upper digestive tract endoscopy using an Olympus Exera II CV 165 endoscope (Olympus Medical System Corporations—Japan, Tokyo). The endoscopic procedures were done by one single endoscopist.

The following endoscopic findings were taken into consideration: chronic and acute gastritis, atrophic gastritis, intestinal metaplasia, gastric polyps, gastro-duodenal ulcer disease and gastric neoplasia.

Gastritis was categorized according to the intensity of mononuclear inflammatory cellular infiltrates, polymorphonuclear activity, atrophy and intestinal metaplasia, while *H. pylori* density was put into mild, moderate and severe categories.

A special emphasis was put on evaluating atrophic gastritis and intestinal metaplasia. In atrophic gastritis, due to the mucosa’s thinning, the submucosal vascular could clearly be seen. In intestinal metaplasia, the endoscopist evaluated the gastric mucosa having a whitish color change with plaques, spots or homogenous discoloration [12].

Aside from intestinal metaplasia and gastric atrophy, polyps of the gastric body and antrum were evaluated. This pathology is frequent in *H. pylori* infection and its eradication can lead to the regression of the polyps [13,14].

### 2.3. Rapid Urease Test (RUT) for the Diagnosis of H. pylori

RUT (AMA Co Ltd., Lehmuskatu, Finland) was applied on all the patients, and only the ones with positive results were included. The RUT is a quick, inexpensive and easy test often used in clinical practice as a screening test for *H. pylori* infection, but not as a gold standard [15].

The RUT is the first-line indication to testing for *H. pylori* in patients that undergo endoscopy and do not have contraindications for biopsies. In order to maximize the test’s sensitivity, the endoscopist should take at least two biopsies, one from the antrum and one from the corpus [16,17]. It has a sensitivity ≥90% and 95–100% specificity according to clinical studies [18].

### 2.4. Histopathology

The biopsy specimens were mainly taken from the antrum, as well as other sites if required. All of the collected biopsies were preserved in 10% neutral formalin and submitted to the Pathology Department of the County Emergency Clinical Hospital of Oradea for paraffin-embedded tissue blocks where 4 mm thick sections were created. For histological analysis, two sets of tissue sections were prepared: one stained with hematoxylin-eosin (H&E), and the other one with Giemsa stain (Epredia-USA; Portsmouth, NH, USA), allowing for the detection of *H. pylori* in the gastric mucosa [19]. The tissue analysis was performed by two skilled pathologists who were double-blinded. The degree of inflammatory mononuclear cellular infiltrates, inflammation activity (neutrophilic infiltrations), glandular atrophy, metaplasia, reparative atypia and dysplasia were all assessed in the biopsies [20].

The severity of mononuclear inflammatory cells infiltrates within the lamina propria was used to grade the cases using the Houston-updated Sydney system, which included absent inflammation (Grade 0), mild inflammation (Grade 1), moderate inflammation (Grade 2) and severe inflammation (Grade 3) [21].

### 2.5. Statistical Analysis

Statistical analysis has been performed using the IBM SPSS Statistic Processor and MedCalc Software Ltd. (Acacialaan, Belgium) [22]. No corrections for multiple comparisons have been applied. Various Fisher exact tests have been performed to determine the correlation between endoscopic measurements vs. histological measurements, wherever corresponding data was available, as well as to verify the correlations with gender and age.

## 3. Results

### 3.1. Patient Characteristics

As the first goal was to highlight infected patients’ features, our study reveals that although most positive patients were male, the percentages were almost equal (47.4% females vs. 52.6% males). Most of them came from urban areas of provenance vs. rural areas (55.6% vs. 44.4%).

### 3.2. Endoscopic Diagnostics

#### Gastric Pathology

The most frequent gastric pathologies associated with *H. Pylori* infection were assessed. By far, chronic gastritis of the antrum was the most common pathology identified in endoscopy (in 79.7% of all evaluated cases), followed by chronic gastritis of the body (in 41.4% of cases). Other diagnosed pathologies were: atrophic gastritis (22.5% of cases), hypertrophic gastritis (22.5% of cases), metaplasia (21.8% of cases), gastric ulcers (in 20.5% of cases), gastric polyps (in 17.4% of cases), acute gastritis (in 5.3% of cases) and gastric neoplasia (in 3.5% of cases). The data are summarized in Table 1.

### 3.3. Histopathology

Gastric biopsies revealed histopathological features of acute gastritis in 31 cases, chronic gastritis in 104 cases, atrophic gastritis in 35 cases, complete/incomplete metaplasia was diagnosed in 23 and 8 cases, respectively, while reduced dysplasia was observed in 20 cases and high-grade dysplasia in 3 cases. Positive Giemsa tests were found in 22 cases. The histopathology diagnostics are summarized in Table 2.

**Figure 1:**
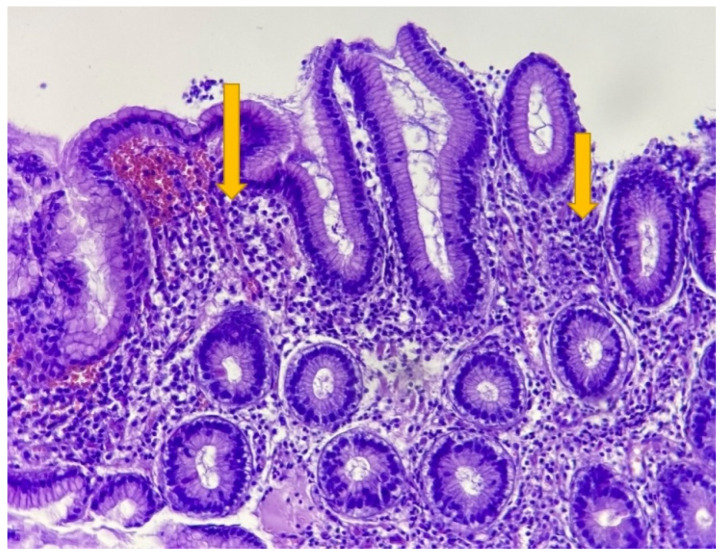
Gastric antral mucosa with massive chronic inflammatory cells: lymphocytes and plasma cells (arrow). H&E 200×.

**Figure 2:**
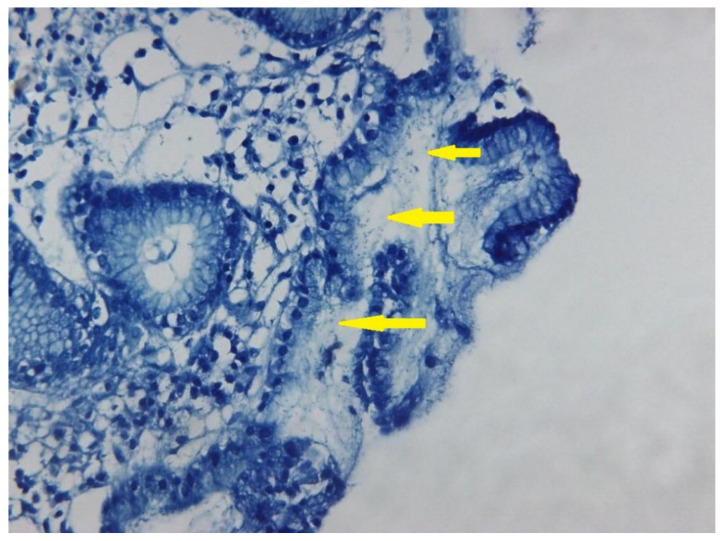
*H. pylori* infection proved by Giemsa stain. Yellow arrows highlight the presence of bacteria in gastric mucosa. 200×.

Relevant histopathological features of different gastric pathologies are presented in Figure 1 and Figure 2, highlighting the value of histopathological diagnostic in the case of *H. pylori* infection. Figure 1 presents an antral gastric mucosa with infiltration of lamina propria by plasma cells/lymphocytes (arrows) arranged as a band-like inflammation. Usually, lymphoid follicles in the antral mucosa can be observed in *H. pylori* infection, when the chronic inflammation is associated with neutrophils, but this feature is not present in the showed image. The tissue examination was performed by considering the parameters: chronic inflammation, activity, atrophy, intestinal metaplasia and the presence of *H. pylori.* As observed in Figure 2, the Giemsa stain improved the *H pylori* diagnostic in the tissue samples. The common shape is a corkscrew, but can be seen also in a coccoid morphology (smaller) in patients with eradication therapy (arrows).

#### Correlation of Endoscopy and Histopathology Results in *H. pylori* Positive Patients

*A*. *Atrophic gastritis*

There were 19 patients diagnosed through endoscopy with corpus atrophic gastritis, out of which 11 patients were females (57.8%) and 8 were males (42.1%). Concerning the place of provenance, most patients came from rural areas (10 patients (52.6%)), while 9 patients came from urban areas (47.3%). In terms of age, elderly patients were more likely to be affected (*p* = 0.024) as there were 14 patients over the age of 65 (73.6%).

Antral atrophic gastritis was diagnosed through endoscopy in 14 cases, out of which 10 patients were females (71.4%), 4 were males (28.6%), 7 came from rural areas and 7 from urban areas. In terms of age, there is no statistical evidence for elderly patients to be more likely affected, as there were 6 patients over 65 years of age (42.8%).

Antrum atrophic gastritis correlated extremely well when measured with both endoscopy and histopathology (Table 3, *p* = 0.001), while corpus atrophic gastritis did not correlate (*p* = 0.4).

*B*. *Gastric metaplasia*

Intestinal metaplasia of the antrum was diagnosed through endoscopy in 23 cases: 13 patients (56.5%) were females and 10 were males (43.5%). Most of them came from urban areas—16 patients (69.5%) and 7 from rural areas (30.4%), without any statistical meaning. Most patients were older than 65 years (13 patients,56.5%).

A statistically significant correlation (*p* = 0.018) between endoscopically produced and histopathology produced diagnostics has been found for antrum metaplasia (Table 3), but not for corpus metaplasia (*p* = 0.12) as a result of the low number of cases.

When combined, the total cases of metaplasia (both corpus and antrum) measured by endoscopy vs. histopathology correlated even better (*p* < <0.001, Table 3), which suggests that the corpus metaplasia data by itself should have also been correlated if not being affected by a lower number of cases.

*C*. *Correlation of corpus atrophy endoscopy with patients’ age*

**Figure 3:**
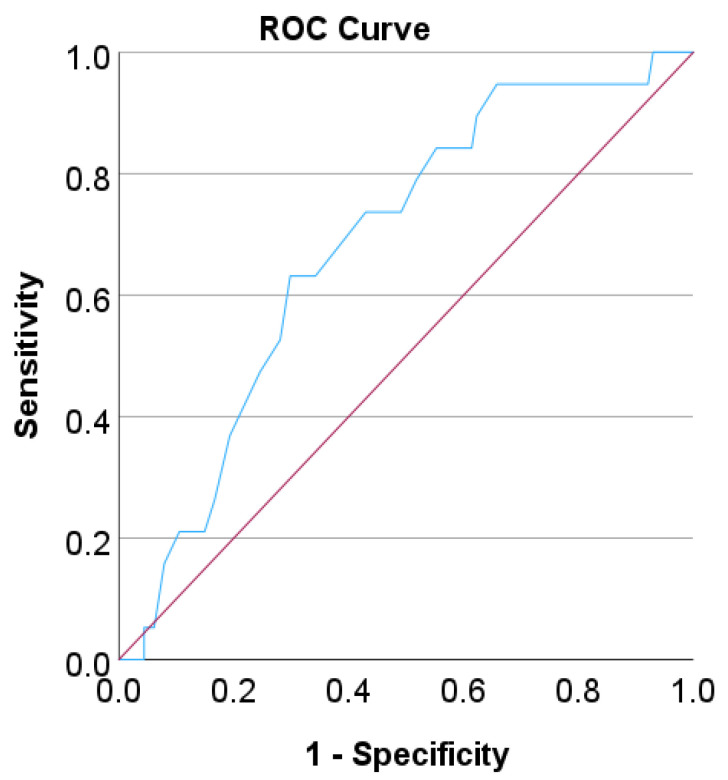
ROC curve analysis for the detection of corpus atrophy based on endoscopy with respect to patients’ age. A diagonal reference is also shown in red.

We performed a ROC curve analysis to find a cut-off age for a classification that gives the best compromise between sensitivity and specificity of predicting corpus atrophy by age when using endoscopy as a detection method (Figure 3). The calculated AUC (area under the curve) was 0.68 (*p* = 0.012).

A cut-off age of 65 leads to a compromise sensitivity of 73% and a specificity of 57%. Fisher’s exact test (two-sided) was performed subsequently, classifying the patients by this cut-off age. According to this test, it was found that endoscopy results for corpus atrophy correlates well with the age of the patients (*p* = 0.024).

*D*. *Gender correlation with endoscopic atrophy and metaplasia*

When using endoscopy, 60% of positively diagnosed patients were females and 40% were males (binomial 95% Cl for the ratio positive males/females ranging from 0.5 to 0.79, average 0.66). We performed the two-sided Fisher exact test to verify the correlation of total atrophy and metaplasia pathologies diagnosed through endoscopy with the patients’ gender. It yielded that females were more likely to be diagnosed with gastric atrophy or intestinal metaplasia (*p* = 0.031).

*E*. *Gastric polyps*

A total of 24 cases of gastric polyps were diagnosed through endoscopy, out of which 13 were located in the corpus and 11 in the antrum. In terms of histopathology, most of them were hyperplastic (62%), followed by inflammatory (31%), adenomatous (3.5%) and dysplastic (3.5%).

Both corpus and antrum polyps diagnosed through endoscopy correlated significantly with the histopathology diagnostics when measured with both methods (*p* < 0.001).

*F*. *Gastric tumors*

A total of four gastric tumors of the corpus were diagnosed through endoscopy, and three through the histopathology method. In this case, the two methods correlated well (*p* = 0.012). All four cases were diagnosed in male patients.

*G*. *Sensitivity and specificity*

Finally, assuming that histology results are always correct, we summarize the sensitivity (true positives divided by all pathologies), specificity (true negatives divided by all non-pathologies), positive predictive value (PPV), the probability that the pathology is present when the test is positive) and negative predictive value (NPV), the probability that the disease is not present when the test is negative) when using endoscopy for all the statistically significant comparisons, as shown in Table 4.

We notice that the specificity when using endoscopy for diagnosis is very high, while the sensitivity is low. Thus, overall, endoscopy is able to identify almost all (92%, CI 88% to 95%) non-pathologies, however it might overdo it and miss a large fraction (45%, CI 34% to 57%) of pathologies. The overall PPV suggests that pathologies detected via endoscopy are likely (64%, CI 53% to 73%) to be real and the overall large percentage of NPV suggests that a particular pathology is very likely absent (88%, CI 85% to 90%) when the endoscopic test result is negative. However, the high value of NPV has to be interpreted with care and patients should not breathe a sigh of relief when the endoscopic result is negative, given the fact that, overall, pathologies represent only 21.3% of the data in Table 4, and endoscopy will miss a large (45%) fraction of pathologies, as shown by the sensitivity.

## 4. Discussion

*H. pylori* is one of the leading factors for gastric neoplasia. The cellular alterations leading to gastric cancer are described by the Correa cascade [23,24,25].

Our study focuses upon the prevalence of these diagnostics in *H. pylori* positive patients. This is important because, in this regard, there are only a few statistical studies focusing on risk factors such as age, gender, place of provenance or association between the endoscopy and histopathology diagnosis.

In this context, our study found that most people affected by *H. pylori* infection were patients over 50 years old (109 patients over 50 years vs. 24 under 50), mostly males (70 males and 63 females) and came from urban areas (74 from urban areas vs. 59 from rural areas). These characteristics are in concordance with other international studies. A complex study conducted in 2019 included 14 controls studies from the Stomach Cancer Pooling (StoP) Project and concluded that men were at higher risk of *H. pylori* infection, while those older than 65 were at higher risk of gastric atrophy [26]. Other studies highlighted the rural area of provenance as a risk factor for *H. pylori* infection [27,28].

Although most cases of premalignant lesions were diagnosed among females (17 cases of metaplasia in females vs. 13 cases in male patients), all gastric tumors were diagnosed in males (4 cases). This means that male patients were more likely to suffer from severe forms of the illness. Our results are consistent with similar international studies showing evidence of the effect of sex hormones on the functioning of the immune system—especially on the balance between pro-inflammatory and regulatory processes and responses [29,30].

On the other hand, the data presented in our study revealed a discrepancy compared to other data reported in the literature regarding the specificity/sensitivity of Giemsa staining. It is well known that the Giemsa stain has a lower sensitivity (60%) compared to hematoxylin-eosin (H&E), but higher specificity (90%), while the false-positive rate is lower compared to H&E [19].

While evaluating the concordance between RUT and Giemsa stain, a percentage of 21% was observed. Generally, clinical studies revealed a high specificity and sensitivity of RUT [18]. A plausible explanation of the false-positive results of RUT is the presence of urease from other bacteria species: *Proteus mirabilis*, *Klebsiella pneumoniae*, *Citrobacter freundii*, *Enterobacter cloacae,* and *Staphylococcus aureus* [31].

However, our results highlighted a clear association between the endoscopic diagnosis of intestinal metaplasia in antrum and histopathologic diagnosis. The same conclusion was true for the atrophic gastritis in the antrum diagnosed by both endoscopy and histopathology. These findings are significant because gastric atrophy and intestinal metaplasia are considered precancerous lesions of the stomach. Previous studies concluded that in terms of histopathology, these lesions are related to gastric cancer, although in low percentage [32].

In Romania, there is a lack of studies conducted on the adult population regarding the correlation between the endoscopic and histopathological appearance of premalignant lesions induced by *H. pylori.* However, in terms of correlation between the endoscopic and histopathological assessment, two studies were conducted on the pediatric population in Cluj Napoca [33] and Targu Mures, Romania [34], with good statistical outputs.

In a tour study, a percentage of 3.5% patients were diagnosed with gastric cancer. When comparing with reported data in the literature, we noticed a large study from Japan, based on simulations, estimating the adjusted cumulative risk of developing gastric cancer due to *H. pylori* for males at 17.03% and for females at 7.66% [35], while another cohort study found the cumulative incidence of cancer to be 0.37%, 0.5% and 0.65%, respectively, after 5, 10 and 20 years from the diagnosis of a *H. pylori* infection [36].

Of course, there are few limitations of our study. First, the data were collected from a single centre and, being a retrospective study, it was difficult to establish cause and effect, and hence, we could not include other risk factors such as smoking, alcohol consumption, non-steroidal anti-inflammatories, a high salt diet, etc. As the data were collected from an endoscopic registry, we were not able to record detailed symptoms, the history of *H. pylori* infection and familial history.

Secondly, we could not compare positive and negative *H. pylori* patients, in order to assess the incidence of premalignant lesions in these groups along with possible statistical correlation between endoscopy and histopathology in negative patients. This item will be our next goal for a future study along with investigating associations between the severity of the illness and hemoglobin levels.

More studies are needed to evaluate the presence of *H. pylori* in biopsies, especially through immunohistochemistry techniques. These techniques possess a very high specificity and sensitivity as they are based on the antigen–antibody reaction.

## 5. Conclusions

Our study provides new insights in terms of the infected *H. pylori* patient profile (mostly elderly males from urban area of provenance). The correlation between endoscopic and histopathological diagnostics was shown to be statistically significant for atrophic gastritis, intestinal metaplasia, gastric polyps and gastric neoplasia. A concordance of only 21% was revealed between Giemsa stain and RUT, highlighting the importance of combining rapid tests with endoscopic and histopathological diagnostic methods for a more accurate, early diagnosis and prevention of gastric cancer.

Moreover, our study highlights the active role of the endoscopist in diagnosing pre-neoplasic gastric lesions such as atrophic gastritis and intestinal metaplasia, especially when biopsies are taken from suspected lesions. It demonstrates the necessity of biopsies and the importance of a more thorough follow-up in order to prevent stomach neoplasia and to establish a better treatment plan.

## Figures and Tables

**Table 1:** Patients’ characteristics and distribution of pathologies as determined by endoscopic investigation.

Demographic Data	Number of Cases/Total/Percentage	
Female	63/133 (47.4%)	
Male	70/133 (52.6%)	
Rural	59/133 (44.4%)	
Urban	74/133 (55.6%)	
Age < 20	0/133 (0%)	
Age 20–29	2/133 (1.5%)	
Age 30–39	5/133 (3.8%)	
Age 40–49	16/133 (12%)	
Age 50–59	24/133 (18%)	
Age 60–69	44/133 (33.1%)	
Age 70–79	36/133 (27.1%)	
Age > 79	5/133 (4.5%)	
Gastric Pathologies (endoscopy)	**Number of cases/total/percentage**	
	**Corpus**	**Antrum**
Atrophic Gastritis	19/133 (14.3%)	14/133 (10.5%)
Acute Gastritis	2/133 (1.5%)	6/133 (4.5%)
Chronic Gastritis	55/133 (41.4%)	106/133 (79.7%)
Hypertrophic Gastritis	13/133 (9.8%)	19/133 (14.3%)
Metaplasia	7/133 (5.3%)	23/133 (17.3%)
Polyps	13/132 (9.8%)	11/133 (8.3%)
Ulcer	10/132 (7.6%)	18/133 (13.5%)
Tumor	4/132 (3.5%)	Not available

**Table 2:** Histopathology diagnostics.

Gastric Pathologies						
	Corpus			Antrum		
Polymorphonuclear cell	15/55 (27.3%)			16/65 (24.6%)		
Lymphoplasmocitar cell	47/55 (85.5%)			57/65 (87.7%)		
Atrophy	19/55 (34.5%)			16/65 (24.6%)		
Complete Metaplasia	4/55 (7.3%)	All Metaplasia	6/55 (10.9%)	19/65 (29.2%)	All Metaplasia	25/65 (38.5%)
Incomplete Metaplasia	2/55 (3.6%)			6/65 (9.2%)		
Reduced Dysplasia	7/55 (12.7%)			13/65 (20%)		
High Dysplasia	1/55 (1.8%)			2/65 (3.1%)		
Hyperplasic Polyps	8/55 (14.5%)	All Polyps	12/55 (21.8%)	10/66 (15.2%)	All Polyps	15/66 (22.7%)
Adenomatous Polyps	0/55 (0%)			1/66 (1.5%)		
Dysplasic Polyps	0/55 (0%)			1/66 (1.5%)		
Inflammatory Polyps	4/55 (7.3%)			5/66 (7.6%)		
Tumor	3/55 (5.5%)			2/66 (3%)		
Acute/(Acute + Chronic)	28/72 (38.9%)					
Giemsa	22/105 RUT positive (21%)					

**Table 3:** Correlations between endoscopy and histopathology.

	Antrum Atrophic Gastritis				Antrum Metaplasia				All Metaplasia			
	Correlation between endoscopy and histopathology in antrum atrophic gastritis				Fisher exact test for the correlation of the endoscopic and histopathology results for metaplasia in antrum				Fisher exact test for the correlation of the endoscopic and histopathology results for all metaplasia (both corpus and antrum).			
		Histopathology		Total		Histopathology		Total		Histopathology		Total
		0	1			0	1			0	1	
Endoscopy	0	46	9	55	0	31	12	43	0	76	16	92
	1	3	7	10	1	9	13	22	1	13	15	28
Total		49	16	65		40	25	65		89	31	129
	*p* = 0.001 *				*p* = 0.018 *				*p* << 0.001 *			

* Statistical significant.

**Table 4:** Sensitivity, specificity, PPV and NPV when using endoscopy as a detection method. CI represents the 95% confidence interval.

	Sensitivity (CI)	Specificity (CI)	PPV	NPV	*p* (Fisher Exact Test)
Antrum Metaplasia	52% (31–72%)	77.5% (62–89%)	59%	72%	0.018 *
All Metaplasia	48% (30–67%)	85% (76–92%)	54%	83%	<<0.001 *
Corpus Polyps	83% (52–98%)	93% (81–99%)	77%	95%	<0.001 *
Antrum Polyps	53% (27–79%)	94% (84–99%)	73%	87%	<0.001 *
Corpus Tumors	67% (9–99%)	96% (87–100%)	50%	98%	0.012 *
Antrum Atrophy	44% (20–70%)	94% (83–99%)	70%	84%	0.001 *
Overall	55% (43–66%)	92% (88–95%)	64%	88%	<<0.001 *

* Statistical significant.

## Data Availability

All data are available in the County Emergency Clinical Hospital of Oradea—Romania, including databases and consultation registers from the Gastroenterology Department, and the paraffin-embedded tissue blocks from the Pathology Department.
